# Poor Sleep and Decision-Making Disturbance Are Associated With Suicidal Ideation in Pre-natal Depression

**DOI:** 10.3389/fpsyt.2021.680890

**Published:** 2021-05-28

**Authors:** Ciqing Bao, Ling Xu, Weina Tang, Shiyu Sun, Wenmiao Zhang, Jincai He, Ke Zhao, Dongwu Xu, Xiaodan Ye

**Affiliations:** ^1^Wenzhou Seventh People's Hospital, Wenzhou, China; ^2^School of Mental Health, Wenzhou Medical University, Wenzhou, China; ^3^Department of Obstetrics, First Affiliated Hospital of Wenzhou Medical University, Wenzhou, China; ^4^Department of Neurology, First Affiliated Hospital of Wenzhou Medical University, Wenzhou, China; ^5^The Affiliated Kangning Hospital of Wenzhou Medical University, Wenzhou, China

**Keywords:** sleep disturbance, antenatal depression, suicidal ideation, decision-making function, pregnancy

## Abstract

Although many risk factors for suicidal ideation have been identified, few studies have focused on suicidal ideation and pre-natal depression. The purpose was to investigate the relationship between decision-making (DM) dysfunction and sleep disturbance on suicidal ideation in pre-natal depression. Participants included 100 women in the third trimester of pregnancy, including pregnant women with pre-natal depression who had recent suicidal ideation (*n* = 30), pre-natal depression without SI (*n* = 35) and healthy controls (*n* = 35). The Iowa Gambling Task (IGT) was used to evaluate the DM function and the Pittsburgh Sleep Quality Index (PSQI) was used to assess the sleep index. The Edinburgh Post-natal Depression Scale (EPDS) was used to assess suicidal ideation and the seriousness of depression. Overall, the two groups with pre-natal depression showed worse sleep quality and decreased DM function compared with healthy controls. The pre-natal depression with suicidal ideation group showed a significantly higher score in subjective sleep quality and a lower score in block 5 of IGT than the pre-natal depression without suicidal ideation group. Further correlation analysis showed that suicidal ideation positively correlated with subjective sleep quality, sleep duration, and daytime function, and negatively correlated with IGT scores. Sleep disturbance and impaired DM function may be risk factors for suicidal ideation in pre-natal depression.

## Introduction

Suicide in the perinatal period is an important public health problem, and suicidal ideation is, one of the most important risk factors for suicidal behavior. During pregnancy, the prevalence of suicidal ideation can reach between 5 and 14% (1–3) and there is a correlation between suicidal ideation and suicide attempts that can result in an elevated suicide risk at some point in the future (1, 4–7). There are many risk factors for suicidal ideation during pregnancy, including accidental pregnancy (8, 9), education level (10, 11), marital status (12, 13), and intimate partner violence (11, 14). Depression and anxiety may be the most important biological factors of perinatal suicide (15, 16). The incidence of perinatal depression is as high as 10–30%, and perinatal depression patients show a higher rate of suicide and self-injury than those with general depression (17). There are few studies on the decision-making (DM) function of perinatal depression patients, even though DM dysfunction may be closely related to suicidal ideation in perinatal depression patients.

DM can be defined as the cognitive process that identifies and selects the best available solution for a given problem or challenging situation based on the value and preferences of the decision maker. The Iowa Gambling Task (IGT) is one of the most commonly used DM tasks (18), and was developed to simulate real-world DM processes in vague and dangerous situations. DM is impaired in various mental illnesses, including obsessive-compulsive disorder (19), schizophrenia (20), and depression (21, 22). Teenagers and adults with DM defects are more likely to display suicidal behavior (23), and more researches have shown that DM play a very important role in suicidal behavior (24). Neurobiological studies have found that the integrity of the orbital cortex/ventral medial PFC (OFC/VMPFC) determines the ability to make decisions. When the OFC/VMPFC is impaired, people suffer from false emotional signals, leading to neglect of unfavorable factors in the future and a focus on immediate interests (25). In depression, structural changes are found in the OFC and VMPFC, and these changes are thought to be closely related to the onset and severity of depression (26, 27).

A large proportion of patients with depression have sleep disturbance and, furthermore, sleep disturbance is more common in perinatal depression than in general depression because of the physiological changes associated with pregnancy (28). There are many studies that show a correlation between pre-natal depression and sleep disturbance (29–33). Jomeen et al. (34) found that about 26% of pregnant women had mild to moderate depressive symptoms, and the sleep quality of the depressed group was worse than that of the non-depressed group. Higher Pittsburgh Sleep Quality Index (PSQI)scores in early pregnancy predict an increase in the Beck depression index scores in the second trimester, and higher PSQI scores in the second trimester predict a higher Beck depression index score in the third trimester, suggesting that sleep quality in the first trimester of pregnancy can predict the clinical symptoms of depression in the third trimester of pregnancy (35). Studies have also shown that there is a correlation between suicidal ideation and sleep during pregnancy (36–38). In one study, in mid-pregnancy, women with high sleep reactivity report elevated symptoms of insomnia, depression, and anxiety, and were more likely to participate in suicidal ideation (39). Previous studies have also suggested that sleep may optimize emotion-guided DM. For example, Pace-Schott et al. reported that after uninterrupted sleep, subjects scores better in the IGT than those that had undergone interrupted sleep (40).

Although there are no studies on DM in pregnant women with pre-natal depression, there has been some research into pregnancy and post-natal cognitive performance and mood. Many studies have shown that there may be cognitive changes in perinatal depression patients (41–46). A Portuguese study found that more dysfunctional beliefs related with maternal responsibility, more frequent negative thoughts related with personal maladjustment and with the metacognitive appraisal of the thoughts' content, and less frequent positive thoughts were significantly associated with postpartum depression symptoms (47). Stress associated with pregnancy may contribute to postpartum mental illness and its associated symptoms by compromising structural plasticity in the mPFC (48). Women may show specific areas of cognitive changes during and after pregnancy, in particular, deficits in verbal learning and memory; mood appears to be impacted as well (46).

There are currently no studies on the association suicidal ideation and DM in patients with perinatal depression. The physiological and psychological mechanisms of high suicidal ideation in perinatal depression are currently unclear. In the present study, we hypothesized that high suicidal ideation in patients with perinatal depression is closely associated with sleep disorders and impaired DM during pregnancy. The purpose of this study was to determine if sleep quality and DM ability are associated with suicidal ideation in pre-natal women with depression compared to pre-natal women without depression.

## Methods

### Participants

A total of 100 pregnant women in late pregnancy were recruited from the obstetrics clinic of the First Affiliated Hospital of Wenzhou Medical University. Three groups were as follows: Group SI included 30 pre-natal depression pregnant women with suicidal ideation (Edinburgh Post-natal Depression Scale (EPDS) >9 and the 10th item in the EPDS except “never”); Group NSI consisted of 35 pregnant women with antenatal depression, but no suicidal ideation (EPDS >9, item 10 in the EPDS was selected as “never”); Group CG was a control group consisting of 35 healthy pregnant women (EPDS ≤ 9, and item 10 in the EPDS was selected as “never”). Inclusion and exclusion criteria were based on clinical structured interviews and self-reports.

The inclusion criteria were: (1) adult (≥18 years old); (2) late pregnancy (28–40 weeks); and (3) willing to sign informed consent.

The exclusion criteria were: (1) history of mental illness or brain disorders; (2) chronic diseases that require regular medical care; (3) intellectual disability; (4) obvious pregnancy complications; and (5) reading and writing dysfunction.

The researchers were composed of psychiatry and mental-health graduate students and physicians, who had undergone consistent training before the experiment began. Our trained researchers collected demographic and clinical information about all pregnant women in face-to-face interviews through standardized questionnaires, including age, height, weight, ethnicity, place of residence, marital status, level of education, monthly family income, history of mental illness, and family history of mental illness. And the standardized questionnaire consists of a demographic scale with consistent guidance and content.

### Assessments

#### The Edinburgh Post-natal Depression Scale

In western countries, the EPDS (49) is a widely used depression assessment scale. And it is believed to be effective in multiple cultures during pregnancy (50–53) and postpartum. It has 10 items testing: mood, pleasure, guilt, anxiety, fear, insomnia, ability to cope, sadness, crying, and self-injury. In accordance with the severity of the relevant symptom, each item is divided into 0–3 points and the total score ranges from 0 to 30 points. The scale has good reliability and validity in populations in mainland China (54). In this experiment, a total score of >9 was classified as depression (55).

#### Suicidal Ideation

To evaluate suicidal ideation, we examined item 10 of the EPDS (presented as “The thought of harming myself has occurred to me”) (56–58). Possible responses included “never” = 0, “hardly ever” = 1, “sometimes” = 2, or “quite often” = 3. Due to the particularity of China's national conditions, “never” is defined as “no suicidal ideation” and the other three are defined as “suicidal ideation.”

#### The Pittsburgh Sleep Quality Index

The PSQI (59) was used to assess sleep quality over the previous month. The maximum total score of the PSQI is 21 points. Nineteen individual items generate seven component scores (range 0–3, with higher scores indicating worse sleep): subjective sleep quality, sleep latency, sleep duration, habitual sleep efficiency, sleep disturbances, use of sleeping medication, and daytime dysfunction. This scale has good internal consistency, test-retest reliability, and validity (60). A score of >5 is indicative of clinically significant sleep disturbance.

#### Iowa Gambling Task

DM was assessed using the IGT (61). Participants completed the computerized version of the IGT consisting of four decks of cards labeled A, B, C, and D. The participants made 100 card selections. After each selection, a specified amount of facsimile money was awarded. At the top of the computer screen, a bar indicates wins and losses and changes according to the amount of money won or lost after each selection. Participants were told that the aim of the game is to win as much money as possible. They were also instructed that some decks were better than others and that they have to avoid the bad decks. The total net score on the IGT is the difference between the total number of cards selected from advantageous decks (decks C′+ D′) and disadvantageous decks (decks A′+B′). Block scores were calculated for every 20 cards selected in the same manner. The total net scores range from −100 to 100 and block scores range from −20 to 20. Positive net and block scores indicate advantageous decision-making.

### Statistical Analysis

Comparison of the three groups (SI, NSI, and CG) with regard to sociodemographic and clinical variables were performed by means of one-way analysis of variance, Kruskal–Wallis test, and the Chi-square test, depending on the variable type (normally distributed, non-normally distributed and categorical, respectively). Checks for deviations from normality were performed by means of the Kolmogorov–Smirnov test. *Post-hoc* pairwise comparisons (by *t*-test, Mann–Whitney *U*-test, or Chi-square test, respectively) were conducted only if the overall group comparison yielded a significant result (*p* < 0.05). In the case of three groups, this sequential testing procedure allowed testing of group differences without adjustment for multiple comparisons.

The same procedure was also used to compare the three groups with PSQI and the DM task IGT. As the IGT consists of five consecutive blocks, we performed an additional repeated-measures analysis of variance with time as a within-subjects factor and group as a between-subjects factor to adjust for potential learning effects.

Spearman correlation analysis was used to analyze the correlations among sleep (PSQI and its seven component scores), DM function, and suicidal ideation in late pregnancy women.

## Results

A total of 100 participants (SI group, *n* = 30; NSI group, *n* = 35; CG group, *n* = 35) were included. Sociodemographic and clinical data are presented in Table 1. The three groups did not differ with respect to age, years of education, marital status, family monthly income, subjective occupational stress, the number of children, or the number of abortions.

**Table 1:** Sociodemographic and clinical variables.

	**Groups**			**Overall comparison**	
	**SI (*n* = 30)**	**NSI (*n* = 35)**	**CG (*n* = 35)**	**Test statistic**	***P*-value**
Age (years)	28.2 (4.0)	29.5 (2.7)	28.8 (4.4)	*F* = 1.16	0.316
Years of education (years)	13.5 (2.0)	12.7 (3.4)	14.0 (2.5)	*F* = 4.81	0.090
%Han	100%	100%	100%	-	-
Marital status				-	-
Married	30 (100%)	35 (100%)	35 (100%)		
The others	0 (0%)	0 (0%)	0 (0%)		
Family monthly income (RMB)				*X*^2^ = 6.24	0.397
<3,000	1 (3.3%)	0 (0%)	1 (2.9%)		
3,000–5,000	5 (16.7%)	12 (34.3%)	6 (17.1%)		
5,000–10,000	11 (36.7%)	14 (40.0%)	17 (48.6%)		
>10,000	13 (43.3%)	9 (25.7%)	11 (31.4%)		
Subjective occupational stress				*X*^2^ = 7.61	0.107
Mild	18 (60.0%)	20 (57.1%)	29 (82.9%)		
Moderately severe	12 (40.0%)	14 (42.9%)	6 (17.1%)		
Number of children				*X*^2^ = 2.63	0.622
0	16 (53.3%)	18 (51.4%)	20 (57.1%)		
1	12 (40.0%)	16 (45.7%)	15 (42.9%)		
2	2 (6.7%)	1 (2.9%)	0 (0.0%)		
Number of abortions				*X*^2^ = 4.27	0.371
0	20 (66.7%)	16 (45.7%)	18 (51.4%)		
1–2	8 (26.7%)	16 (45.7%)	16 (45.7%)		
≥3	2 (6.7%)	3 (8.6%)	1 (2.9%)		

*SI, Suicidal ideation group; NSI, Non-suicidal ideation group; CG, Control group; RMB, Chinese legal currency Age was analyzed by one-way analysis of variance, all other continuous variables by Kruskal-Wallis test, all categorical variables by Chi-square test*.

Table 2 shows the results of the EPDS scores, EPDS scores without suicidal ideation, and PSQI between groups. Compared with the NSI group, the EPDS scores of the SI group were significantly increased, which was due to the higher score of the suicide ideation factor on the EPDS. There were significant differences between the three groups except for the PSQI scale and the EPDS scale without suicidal factor scores. There were also significant differences in subjective sleep quality, sleep disturbance, and daytime function in the PSQI scale. For the subjective sleep quality, the SI group showed a significantly higher score than the other two groups. This means that women with suicidal ideation had worse subjective sleep quality than women without suicidal ideation and healthy controls. No significant differences were found between the NSI and CG groups. The scores of sleep disturbances and daytime function in the SI group were significantly higher than those in the CG group.

**Table 2:** Differences in scores of PSQI and EPDS among the three groups (SI, NSI, CG).

	**Groups**			**Group comparison**			
				**Overall**	**Pairwise**		
	**SI (*n* = 30)**	**NSI (*n* = 35)**	**CG (*n* = 35)**	***P*-value**	**SI vs. NSI *P*-value**	**SI vs. CG*P*-value**	**NSI vs. CG *P*-value**
EPDS (SD)	13.57 (3.4)	11.63 (2.1)	5.6 (2.2)	<0.001	0.018	<0.001	<0.001
EPDS without suicidal ideation (SD)	12.27 (3.5)	11.63 (2.1)	5.6 (2.2)	<0.001	0.535	<0.001	<0.001
PSQI (SD)	9.27 (4.1)	7.66 (3.5)	5.97 (2.6)	0.002	0.074	0.001	0.048
Subjective sleep quality (SD)	1.67 (0.7)	1.31 (0.6)	1.14 (0.4)	0.005	0.05	0.001	0.185
Sleep latency (SD)	1.87 (0.9)	1.57 (1.0)	1.31 (0.8)	0.074	-	-	-
Sleep duration (SD)	0.87 (1.1)	0.54 (1.0)	0.31 (0.7)	0.091	-	-	-
Habitual sleep efficiency (SD)	1.13 (1.2)	0.89 (1.1)	0.80 (0.9)	0.671	-	-	-
Sleep disturbances (SD)	1.67 (0.6)	1.54 (0.5)	1.31 (0.4)	0.05	0.541	0.024	0.055
Use of sleeping medication (SD)	0	0	0	1.00	-	-	-
Daytime dysfunction (SD)	2.0 (0.9)	1.8 (0.7)	1.0 (0.9)	<0.001	0.161	<0.001	0.001

*SI, Suicidal ideation group; NSI, Non-suicidal ideation group; CG, Control group; EPDS, The Edinburgh Post-natal Depression Scale; EPDS without suicidal ideation. The sum of the scores of the remaining nine items except the 10th item of EPDS; PSQI, The Pittsburgh Sleep Quality Index; Subjective sleep quality, sleep latency, sleep duration, habitual sleep efficiency, sleep disturbances, use of sleeping medication, and daytime dysfunction are the seven sub-items of PSQI*.

There were significant differences of in the IGT scores and block3-block5 scores among the groups (Table 3). Among these differences, the block 5 group were significant after comparison. Repeated measures ANOVA revealed significant positive effects in the different groups of IGT (*F* = 10.653, *p* < 0.001) and significant learning effects from the first to fifth blocks of the IGT (*F* = 17.905, *p* < 0.001). In addition, test time interacted with the group (*F* = 3.706, *P* = 0.001).

**Table 3:** Differences in decision-making functions among three groups (SI, NSI, CG).

	**Groups**			**Group comparison**			
				**Overall**	**Pairwise**		
	**SI (*n* = 30)**	**NSI (*n* = 35)**	**CG (*n* = 35)**		**SI vs. NSI**	**SI vs. CG**	**NSI vs. CG**
				***P*-value**	***P*-value**	***P*-value**	***P*-value**
IGT (SD)	−15.13 (38.0)	−9.94 (21.2)	10.74 (27.2)	<0.001	0.191	<0.001	0.001
Block1 (SD)	−4.1 (7.0)	−4.2 (6.9)	−3.1 (6.2)	0.803	0.959	0.554	0.494
Block2 (SD)	−4.6 (9.0)	−3.6 (6.3)	−1.5 (8.5)	0.186	0.574	0.150	0.240
Block3 (SD)	−5.4 (8.4)	−2.2 (6.5)	2.7 (7.6)	<0.001	0.089	<0.001	0.005
Block4 (SD)	−2.6 (8.0)	−2.2 (7.0)	5.5 (8.1)	<0.001	0.855	<0.001	<0.001
Block5 (SD)	−2.3 (8.8)	2.3 (6.9)	7.1 (7.8)	<0.001	0.022	<0.001	0.008

*IGT, Iowa gambling task; SI, Suicidal ideation group; NSI, Non-suicidal ideation group; CG, Control group*.

Spearman correlation analysis (Table 4) showed that suicide ideation positively correlated with pre-natal depression (*r* = 0.415, *P* < 0.001), PSQI (*r* = 0.289, *P* = 0.004), subjective sleep quality (*r* = 0.305, *P* = 0.002), sleep duration (*r* = 0.212, *P* = 0.034), and daytime dysfunction (*r* = 0.299, *P* = 0.002), and negatively correlated with IGT total score (*r* = −0.265, *p* = 0.008).

**Table 4:** Spearman correlation matrix of suicidal ideation, PSQI and IGT.

	**Suicidal ideation**	**EPDS without suicidal ideation**	**IGT**	**PSQI**	**Subjective sleep quality**	**Sleep duration**	**Daytime dysfunction**
**Suicidal ideation**	–	0.415^***^	−0.265^**^	0.289^**^	0.305^**^	0.212^*^	0.299^**^
**EPDS without suicidal ideation**		-	−0.365^***^	0.351^***^	0.251^*^	0.131	0.391^***^
**IGT**			–	−0.207^*^	−0.123	−0.123	−0.329^**^
**PSQI**				–	0.710^***^	0.707^***^	0.634^***^
**Subjective sleep quality**					–	0.444^***^	0.521^***^
**Sleep duration**						–	0.288^**^
**Daytime dysfunction**							–

*Spearman correlation*

* *p < 0.05*,

** *p < 0.01*,

*** *p < 0.001*.

## Discussion

To the best of our knowledge, this is the first study to assess DM and sleep in pre-natal depression patients with suicidal ideation. The risk-decision made by the suicidal ideation group in the fifth block of the IGT was significantly higher than that of the NSI group and the healthy control group, which indicates that people with DM defects have stronger suicidal ideation. Suicide ideation positively correlated with pre-natal depression, subjective sleep quality, sleep duration, and daytime dysfunction, and negatively correlated with DM function. The three groups in our study did not differ significantly in social demographics.

Our findings are generally consistent with the existing literature documenting the association of poor subjective sleep quality with the likelihood of suicidal ideation among men and non-pregnant women (62, 63). However, there are few studies on sleep and pre-natal depression and suicidal ideation during pregnancy. Among them, one study showed that poor subjective sleep quality was associated with an increased likelihood of suicidal ideation (36). The article mentioned that after adjusting for depression, the probability of suicidal ideation increases by 18% for every unit increase in the global PSQI score. The proportion of subjective sleep quality and suicidal ideation in patients with depression increased by 3.5-fold compared with patients without risk factors. Another study published in 2016 showed similar results (37).

Our study confirmed the correlation between suicidal ideation and subjective sleep quality, and the difference was also significantly related to sleep duration and daytime dysfunction. The causal relationship between sleep disorders and suicide in major depression is unclear, however, there is evidence that the activities of the three neurobiological systems play a key role in the pathophysiology of suicidal behavior, i.e., hyperactivity of the hypothalamus-pituitary-adrenal axis, dysfunction of the serotoninergic system and overactivity of the noradrenergic system. Not only the first and last systems appear to be involved in the response to stress events, the hyperactivity of the HPA axis and the dysfunction of the serotonergic system are also associated with changes in the sleep-wake cycle, such as insomnia (64–67). In depression, HPA hyperactivity can have many negative effects on sleep. It can lead to sleep fragmentation, decreased slow-wave sleep, and shortened sleep time (66). It can therefore be assumed that neurobiological dysfunction mediates the occurrence of suicidal behavior through the disturbed regulation of basic neuropsychological functions. The main clinical significance of suicidal ideation/sleep disturbance is that sleep assessment may help assess the risk of suicide in patients with antenatal depression.

Previous studies of DM and suicidal ideation have produced controversial results. Some studies have shown that IGT scores are significantly lower in people who currently have suicidal ideation compared with healthy controls (68). However, in contrast, some studies found no association between suicidal ideation and DM (69, 70). DM deficiencies were related to suicide attempts in adolescents and adults, and the group differences in DM did not persist after controlling for current emotional problems and the use of psychotropic substances. In this study, there was no significant difference in the IGT net score between the SI group and the NSI group, but there was a significant difference compared with the healthy control, indicating that there was a decision deficit in the pre-natal depression groups with or without suicidal ideation. By comparing the net scores and their trends in different blocks, we analyzed the DM characteristics and strategy adjustment of the subjects in the income-loss situation (71). There was no significant difference in the net scores of blocks 1–2 between the three groups in this study. In blocks 3–4, both groups with depression had significantly lower scores than the healthy controls, while there was no significant difference between the SI and NSI groups. The two groups of pregnant women with pre-natal depression showed no difference in performance at the beginning and the middle stage. As the task progressed, the net scores of the two groups in the fifth block were different. The SI group scores were significantly lower than those in the NSI group, and the scores of both groups were lower than those of the healthy control group. These data shows that patients with pre-natal depression lack decision-making ability, have poor strategy adjustment, and do not show the “learning effect” of normal healthy people or improve the net score, while the DM function defects of pre-natal depression patients with suicidal ideation are more serious.

A previous study suggested that IGT performance was not mediated by the conscious knowledge of risk. Patients selected cards that brought higher immediate rewards despite more severe penalties, and patients with OFC/VMPFC lesions often showed this pattern of behavior (25, 72). Another study found that REM sleep in emotion-guided decision making plays a selective role, the subjects performed highly on the IGT, whose OFC/VMPFC regions were selectively activated in REM sleep (73). Sleep disturbances, including insomnia and sleep deprivation, can produce a labile mood (74), which may correspond with neural responses that suggest abnormal reward processing and autonomic hyperarousal (75), limbic overactivity (76), and loss of medial pre-frontal cortex connectivity (77). Having sleep disturbance could worsen the altered reward processes in mothers with depression and SI (78). We hypothesized that sleep inefficiency and daytime dysfunction would aggravate impairment of DM function in patients and the correlation results support our hypothesis.

Our study shows that suicidal ideation and depressive symptoms of pregnant women need to be carefully monitored, especially in those with poor sleep quality. Pregnant women often report poor sleep quality and those with poor subjective sleep quality should be evaluated for suicide ideation. Actively dealing with sleep problems may reduce suicide behavior of pregnant women. For example, investigators have noted that behavioral (e.g., sleep hygiene, stimulus control, and imagery rehearsal therapy) and pharmacological interventions may be particularly promising modalities for reducing the risk of suicidal ideation (79). This study suggests that the decision-making function impairment may be associated with pre-natal depression and suicidal ideation in pregnant women, However, further studies on the neuropathological mechanism are needed, such as functional magnetic resonance imaging (fMRI).

This study reports the DM profile of recent pre-natal depression subjects with suicidal ideation and confirms the existence of decision deficits in this population. The study also found correlations between antenatal depression, suicidal ideation and sleep disturbance. There is an urgent need to raise awareness of this issue and further longitudinal studies should be encouraged to examine other cognitive functions that may be related to suicide status.

There are several limitations in our study. Firstly, the sample size was relatively small (*n* = 100) and it will be necessary to study a larger sample size to reduce the type II error rate. Secondly, due to the nature of cross-sectional studies, we are not sure of the dynamic relationship between suicidal ideation and sleep in perinatal depression patients during pregnancy, nor can we infer the causal relationship between suicidal ideation and impaired DM function. Longitudinal studies are needed to address these questions and verify our findings. Thirdly, In this study, only the subjective scale (PSQI) was used to evaluate sleep, without using objective evaluation indicators, such as polysomnography test. Finally, due to the absence of brain imaging, in this study we do not know whether any neuroanatomical/functional changes in the patients' brains occurred.

## Data Availability Statement

The data analyzed in this study is subject to the following licenses/restrictions: Due to relevant data protection laws. Requests to access these datasets should be directed to baociqing@163.com.

## Ethics Statement

The studies involving human participants were reviewed and approved by Ethics Committee of Wenzhou Medical University. The patients/participants provided their written informed consent to participate in this study.

## Author Contributions

CB and KZ conceived and designed the study and developed it in discussion with LX, WT, SS, and WZ. SS, WT, and LX were involved in the acquisition and analysis of the data. DX and XY provided research funding. CB wrote the first draft of the article. All authors participated in the interpretation of the data, contributed to critically revising the paper, read, and approved the final manuscript.

## Conflict of Interest

The authors declare that the research was conducted in the absence of any commercial or financial relationships that could be construed as a potential conflict of interest.

## Abbreviations

DM: decision-making
IGT: Iowa Gambling Task
PSQI: Pittsburgh Sleep Quality Index
EPDS: Edinburgh Post-natal Depression Scale
OFC: orbitofrontal cortex
VMPFC: ventromedial PFC.
